# SARS-CoV-2 viral RNA load dynamics in the nasopharynx of infected children

**DOI:** 10.1017/S095026882100008X

**Published:** 2021-01-11

**Authors:** K. Q. Kam, K. C. Thoon, M. Maiwald, C. Y. Chong, H. Y. Soong, L. H. Loo, N. W. H. Tan, J. Li, K. D. Nadua, C. F. Yung

**Affiliations:** 1 Infectious Disease Service, Department of Paediatrics, KK Women's and Children's Hospital, Singapore; 2 Duke-NUS Graduate Medical School, Singapore; 3 Yong Loo Lin School of Medicine, National University of Singapore, Singapore; 4 Lee Kong Chian School of Medicine, Imperial College London, Nanyang Technological University, Singapore; 5 Department of Pathology and Laboratory Medicine, KK Women's and Children's Hospital, Singapore; 6 Department of Microbiology and Immunology, National University of Singapore, Singapore

**Keywords:** Children, COVID-19, cycle threshold, paediatric, SARS-CoV-2, viral load

## Abstract

It is important to understand the temporal trend of the paediatric *severe acute respiratory syndrome coronavirus 2* (SARS-CoV-2) viral load to estimate the transmission potential of children in schools and communities. We determined the differences in SARS-CoV-2 viral load dynamics between nasopharyngeal samples of infected asymptomatic and symptomatic children. Serial cycle threshold values of SARS-CoV-2 from the nasopharynx of a cohort of infected children were collected for analysis. Among 17 infected children, 10 (58.8%) were symptomatic. Symptomatic children, when compared to asymptomatic children, had higher viral loads (mean cycle threshold on day 7 of illness 28.6 *vs*. 36.7, *P* = 0.02). Peak SARS-CoV-2 viral loads occurred around day 2 of illness in infected children. Although we were unable to directly demonstrate infectivity, the detection of significant amount of virus in the upper airway of asymptomatic children suggest that they have the potential to shed and transmit SARS-CoV-2. Our study highlights the importance of contact tracing and screening for SARS-CoV-2 in children with epidemiological risk factors regardless of their symptom status, in order to improve containment of the virus in the community, including educational settings.

The viral load of the novel coronavirus, *severe acute respiratory syndrome coronavirus 2* (SARS-CoV-2), has been reported to peak within the first week of disease in throat and sputum samples in the adult population [1, 2]. Virus RNA was also detectable in some asymptomatic adult patients, suggesting viral shedding and potential for transmission during the asymptomatic period [2–4]. A proportion of coronavirus disease 2019 (COVID-19)-infected children are known to be asymptomatic or have mild-to-moderate disease [5–7]. Previously, we reported a paucisymptomatic SARS-CoV-2-infected infant who presented with high viral load prior to symptom onset, viral shedding up to day 18 of illness and significant environmental viral contamination [8, 9]. A recent study in Korea also suggests that SARS-CoV-2 can be detected in children for a mean of more than 2 weeks and a proportion of the infected children are asymptomatic despite high viral load [10]. There is a need to improve the understanding of the viral load dynamics of SARS-CoV-2 in infected children so as to postulate the role of children in the transmission of COVID-19 in schools and the community. In this study, we analysed the daily trends of SARS-CoV-2 viral load from nasopharyngeal samples of infected symptomatic and asymptomatic paediatric patients.

KK Women's and Children's Hospital (KKH) is an 830-bed hospital that provides care for approximately 500 children's emergency daily attendances and 12 000 deliveries per year. It is the primary hospital for evaluation and isolation of COVID-19 in the paediatric population in Singapore. A line list of confirmed paediatric cases who presented to our institution from 23 March 2020 to 5 April 2020 was extracted from hospital records. Confirmed cases that were diagnosed by positive SARS-CoV-2 PCR from nasopharyngeal swabs using the real-time reverse transcription polymerase chain reaction (rRT-PCR) for the E gene were included. Nasopharyngeal swabs were taken daily or every other day from the confirmed cases (supplementary table S1). A cycle threshold (Ct) of 45 is considered to be undetectable for the virus. Age, gender and the Ct values of all nasopharyngeal swabs for SARS-CoV-2 for each child were also obtained. The symptoms of the infected children were verified through history taking. Asymptomatic disease was defined as having a positive nasopharyngeal SARS-CoV-2 PCR test without any fever, respiratory or gastrointestinal symptoms. The asymptomatic infected children were detected through contact tracing as they were in close contact with household SARS-CoV-2-infected adults. Contact tracing was done by trained personnel from the Singapore Ministry of Health and these children were conveyed to our institution for admission when the SARS-CoV-2 PCR test was positive. In our institution, children were considered to have recovered from COVID-19 and were discharged from the hospital when they had negative SARS-CoV-2 PCR results from two nasopharyngeal swabs on consecutive days.

The Ct values were reported in relation to the day of illness or day of diagnosis for symptomatic and asymptomatic patients, respectively (Fig. 1A). Nasopharyngeal swabs were collected using Mini UTM Kits (Copan, Brescia, Italy) with flocked swabs and 1 ml of universal transport medium. From this medium, 200 μl was used for extraction of viral nucleic acids using the EZ1 Virus Mini Kit v2.0 (Qiagen, Hilden, Germany) into 60 μl of eluate. rRT-PCR targeting the SARS-CoV-2 E gene was performed according to the method by Corman *et al*. [11]. All reactions were run on a QuantStudio 5 instrument (ThermoFisher Applied Biosystems, Foster City, CA, USA). A volume of 5 μl was used in PCRs, and with conversion factors, this represented 1/60th of the swab contents per reaction. The positive control consisted of a plasmid with a SARS-CoV-2 E gene insert, adjusted to 1000 copies per reaction. During the study period, the mean Ct value of all positive control PCRs was 29.86 and the standard deviation was ± 0.68. Thus, using a conversion factor (1/60 of the swab content per reaction), a Ct value of 29.86 corresponded to approximately 6 × 10^4^ (1000 × 60) virus genome copies per swab. All first-time positive results for each individual patient were confirmed by a second PCR assay, the Fortitude PCR kit (A*Star, Singapore) on a CFX96 thermocycler (Bio-Rad, Hercules, CA, USA).

**Fig. 1.**
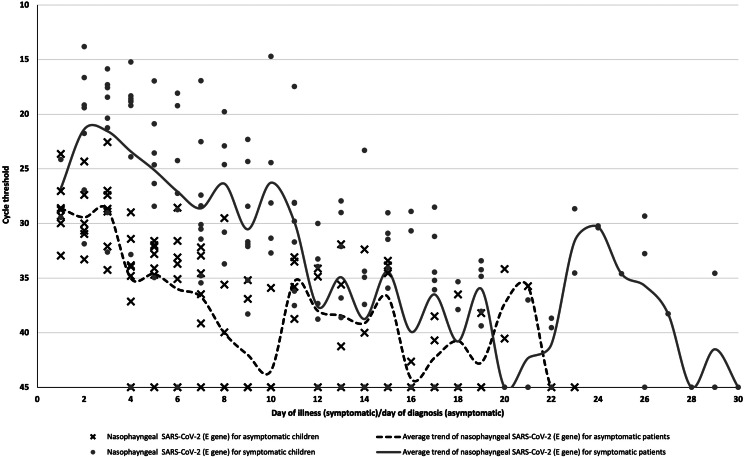
SARS-CoV-2 temporal viral load dynamics in the nasopharynx of paediatric COVID-19 patients, Plotted against day of illness (symptomatic children) or day of diagnosis (asymptomatic children). SARS-CoV-2: *severe acute respiratory syndrome coronavirus 2.*

Continuous variables that were normal in distribution were expressed as mean (standard deviation (s.d.)) and compared with two samples *T* test. Continuous variables that were not normal in distribution were expressed as median (range). Categorical variables were expressed as numbers (%). A two-sided *α* of less than 0.05 was considered statistically significant. Statistical analyses were carried out using the SPSS software, version 23 (IBM, Armonk, NY, USA). The average Ct values for each day of illness and diagnosis were obtained for the asymptomatic and symptomatic children. The graphs were drawn to compare the average Ct trends on each day of illness/diagnosis between the two groups. The study was approved by the institutional ethics review board. Written informed consent was waived in light of the need to inform public health outbreak control policies.

From 23 March to 5 April 2020, 17 children with confirmed COVID-19 via rRT-PCR E gene assay were cared for as inpatients at our institution. The median age of the cohort was 7.7 years old (range: 0.3−15.8 years old). In our cohort, 10 (58.8%) of the children were symptomatic. All symptomatic children had a mild illness with upper respiratory tract infection and their symptoms resolved by day 5 of illness. No complications or evidence of pneumonia were observed. All asymptomatic children remained well with no development of fever, respiratory or gastrointestinal symptoms until discharge.

The mean duration of viral shedding was about 16 days (s.d.: 7.4 days) in our cohort and the longest duration of viral shedding was 30 days in a symptomatic child. Symptomatic children had longer durations of viral shedding but this was not statistically significant (mean 17 days *vs*. 14 days, *P* = 0.48). Higher viral loads were observed in symptomatic children when compared to asymptomatic children (mean cycle threshold on day 7 of illness 28.6 *vs*. 36.7, 95% CI = 1.9−14.3, *P* = 0.02).

The highest SARS-CoV-2 viral loads occurred around day 2 of illness in symptomatic children or days of diagnosis in asymptomatic children, respectively (Fig. 1A). However, our findings are limited by the lack of data from day 1 of illness for most symptomatic children. The detection of early viral peaks in the initial stage of illness in our paediatric cases concurs with data from other studies [12–14]. This raises the possibility that children with COVID-19 may transmit the virus to others during the early stage of the illness. Although we were unable to establish if the virus was already detectable in high viral loads in the pre-symptomatic phase in our cohort of patients, the high viral load in our symptomatic children in the very early stage of illness implies the transmission potential of pre-symptomatic children if they are not identified and isolated in the pre-symptomatic phase.

Our findings revealed that symptomatic COVID-19-infected children may have higher viral loads in the initial stage of illness than asymptomatic children. Han *et al*. [13] reported similar findings from a smaller cohort in South Korea, but daily swab data were not available. This suggests that symptomatic children may have a higher risk of transmitting the virus than asymptomatic children. These findings could be used to guide modelling work to help understand the role of children in driving transmission in schools and the wider population, in order to inform public health interventions including future vaccination policies.

We found that the majority of patients (15 out of 17, 88.2%) had detectable virus even on day 7 of illness/diagnosis and the mean duration of viral shedding was 16 days. If testing resources need to be preserved, we suggest that infected children do not need to be tested for the virus for at least the first 7 days of illness/diagnosis to confirm they no longer need to be isolated unless clinically indicated. Even though both symptomatic and asymptomatic patients displayed long durations of detectable virus via PCR testing on nasopharyngeal specimens, the patients might not be infectious. Wölfel *et al*. [15] found that the viral culture was negative after day 8 of symptom onset despite detectable SARS-CoV-2 PCR from the respiratory specimens. Hence, detectable viral PCR may not equate to transmissibility. Isolation of paediatric COVID-19 cases to prevent ongoing transmission may need to continue for at least 1−2 weeks. Children with epidemiological links to confirmed cases in the household and schools should be placed under quarantine for a least 2 weeks, as these children may shed the virus during the asymptomatic or pre-symptomatic phase.

Although our sample size is limited, frequent nasopharyngeal specimens were taken for every case which accounted for individual level variations in viral shedding patterns in infected children. Our PCR assay was not set up to be exactly quantitative, but approximate viral loads can be gleaned from Ct values, using the dilution factors and the fact that positive controls contained 1000 copies of a plasmid per reaction. As per all observational studies, it is difficult to ascertain the exact day of acquisition of the virus, especially for asymptomatic infected children. Lastly, we did not do viral culture on the nasopharyngeal specimens, and thus viral transmissibility has not been shown.

In conclusion, our study found that symptomatic infected children have higher viral RNA loads than asymptomatic children during the initial stage of the disease. Peak SARS-CoV-2 viral loads occurred in the early stages of illness in infected children. Hence, symptomatic SARS-CoV-2-infected children may have higher transmissibility than asymptomatic children. These findings highlights the importance of screening for SARS-CoV-2 in children with epidemiological risk factors, particularly symptomatic children, in order to improve containment of the virus in the community, including educational settings.

## Supplementary material

For supplementary material accompanying this paper visit https://doi.org/10.1017/S095026882100008X. click here to view supplementary material

## Data

The readers can contact the corresponding author if they want access to the resources and data necessary to replicate the findings discussed in this paper.
