# Supplementary material for: SARS-CoV-2 viral RNA load dynamics in the nasopharynx of infected children
Source: Epidemiol Infect. 2021 Jan 11;149:e18. doi: 10.1017/S095026882100008X (PMC7847743; doi:10.1017/S095026882100008X)
Supplement: Supplementary file 1 [file S095026882100008Xsup001.docx]

**EPIDEMIOLOGY AND INFECTION**

**SUPPLEMENTARY MATERIAL**

**SARS-CoV-2 Viral RNA Load Dynamics in the Nasopharynx of Infected Children**

K. Q. KAM ^1-3^, K. C. THOON ^1-4^, M. MAIWALD ^2, 5, 6^, C. Y. CHONG ^1-4^, H. Y. SOONG ^5^, L. H. LOO ^5^, N. W. H. TAN ^1-4^, J. LI ^1-3^, K. D. NADUA ^1-3^, C. F. YUNG ^1, 2, 4^

^1^Infectious Disease Service, Department of Paediatrics, KK Women’s and Children’s Hospital, Singapore

^2^Duke-NUS Graduate Medical School, Singapore

^3^Yong Loo Lin School of Medicine, National University of Singapore, Singapore

^4^Lee Kong Chian School of Medicine, Imperial College London, Nanyang Technological University, Singapore

^5^Department of Pathology and Laboratory Medicine, KK Women’s and Children’s Hospital, Singapore

^6^Department of Microbiology and Immunology, National University of Singapore, Singapore

| Patient^ | Nasopharyngeal cycle threshold value* | | | | | | | | | | | | | | | | | | | | | | | | | | | | | |
| --- | --- | --- | --- | --- | --- | --- | --- | --- | --- | --- | --- | --- | --- | --- | --- | --- | --- | --- | --- | --- | --- | --- | --- | --- | --- | --- | --- | --- | --- | --- |
|  | **Day of illness/day of diagnosis** | | | | | | | | | | | | | | | | | | | | | | | | | | | | | |
|  | **1** | **2** | **3** | **4** | **5** | **6** | **7** | **8** | **9** | **10** | **11** | **12** | **13** | **14** | **15** | **16** | **17** | **18** | **19** | **20** | **21** | **22** | **23** | **24** | **25** | **26** | **27** | **28** | **29** | **30** |
| 1 | 23.6 | 24.3 | 22.6 | 31.4 | 32.0 | 35.1 | 32.2 | 35.6 | 36.9 | 45.0 | 38.8 | 45.0 | 41.3 | 45.0 | 34.6 | 45.0 | 38.5 | 45.0 | 45.0 |  |  |  |  |  |  |  |  |  |  |  |
| 2 | 30.0 | 30.7 | 32.1 | 34.8 | 31.6 | 33.1 | 39.2 | 29.5 | 45.0 | 45.0 |  |  |  |  |  |  |  |  |  |  |  |  |  |  |  |  |  |  |  |  |
| 3 | 29.3 | 31.0 | 28.6 | 45.0 | 34.7 | 45.0 | 33.0 | 45.0 | 45.0 | 45.0 | 33.1 | 34.9 | 35.6 |  | 33.4 |  | 40.7 |  | 45.0 | 34.2 |  | 45.0 | 45.0 |  |  |  |  |  |  |  |
| 4 | 28.6 | 27.4 | 27.0 | 29.0 | 32.8 | 31.6 | 45.0 | 40.0 | 45.0 | 45.0 |  |  |  |  |  |  |  |  |  |  |  |  |  |  |  |  |  |  |  |  |
| 5 | 28.8 | 33.3 | 34.2 | 34.0 | 34.1 | 45.0 | 34.6 | 45.0 | 45.0 | 45.0 |  |  |  |  |  |  |  |  |  |  |  |  |  |  |  |  |  |  |  |  |
| 6 | 33.0 | 30.0 | 28.9 | 37.2 | 32.1 | 28.6 | 36.5 |  |  | 35.9 | 33.5 | 34.1 | 31.9 | 32.4 | 45.0 | 42.6 | 45.0 | 36.5 | 38.2 | 40.6 | 35.8 |  |  |  |  |  |  |  |  |  |
| 7 | 27.1 |  | 27.4 | 33.8 | 45.0 | 33.7 |  | 45.0 | 35.2 |  | 35.8 |  | 45.0 | 40.0 | 33.8 | 45.0 | 45.0 |  |  |  |  |  |  |  |  |  |  |  |  |  |
| 8 |  | 19.4 | 21.2 | 18.7 | 26.4 | 27.2 | 30.5 | 30.8 |  | 28.1 |  | 33.3 |  | 34.9 |  | 45.0 | 35.2 | 37.9 | 38.0 | 45.0 | 37.0 | 38.7 | 28.7 | 30.2 |  | 45.0 | 38.3 |  | 45.0 | 45.0 |
| 9 |  |  |  | 23.9 | 23.5 | 24.2 | 30.1 | 33.7 | 24.3 | 32.7 | 31.7 | 45.0 | 29.0 | 37.4 | 30.9 | 30.7 | 45.0 | 45.0 |  |  |  |  |  |  |  |  |  |  |  |  |
| 10 |  | 19.2 | 18.4 | 15.2 | 20.9 | 19.2 | 27.4 | 24.6 | 31.7 | 31.4 |  | 30.0 |  | 45.0 | 34.1 |  | 34.5 |  | 33.4 |  | 45.0 | 39.6 |  | 30.4 |  | 29.3 |  | 45.0 | 45.0 |  |
| 11 |  |  | 17.3 | 18.3 | 16.9 | 18.1 | 31.4 | 22.9 | 22.3 | 24.4 | 28.2 |  | 27.9 |  | 29.0 |  | 31.2 |  | 34.2 |  |  | 45.0 | 34.5 |  | 34.6 | 32.8 |  |  | 34.6 |  |
| 12 |  | 26.9 | 32.6 | 45.0 |  | 45.0 |  |  |  |  |  |  |  |  |  |  |  |  |  |  |  |  |  |  |  |  |  |  |  |  |
| 13 |  |  |  |  |  | 28.7 | 16.9 | 19.8 | 31.9 | 14.7 | 17.5 |  |  | 23.3 |  | 28.9 | 28.5 | 35.3 | 34.9 | 45.0 | 45.0 |  |  |  |  |  |  |  |  |  |
| 14 |  | 13.8 | 15.9 | 18.6 | 28.4 |  | 28.4 |  | 35.2 |  | 37.5 | 38.8 | 38.6 | 45.0 | 31.5 | 45.0 | 45.0 |  |  |  |  |  |  |  |  |  |  |  |  |  |
| 15 |  | 16.7 | 17.6 | 18.9 |  |  | 22.5 |  | 28.4 |  | 28.1 | 33.9 | 45.0 | 45.0 |  |  |  |  |  |  |  |  |  |  |  |  |  |  |  |  |
| 16 | 29.5 | 31.9 | 29.0 | 32.9 | 34.9 |  | 34.8 |  | 32.1 |  | 36.2 | 45.0 | 36.8 | 45.0 | 35.9 | 45.0 | 36.1 | 45.0 | 39.4 |  |  |  |  |  |  |  |  |  |  |  |
| 17 | 24.1 | 21.8 | 20.4 | 19.2 | 24.6 |  | 35.4 |  | 38.3 |  | 29.8 | 37.3 | 32.1 | 34.4 | 45.0 | 45.0 |  |  |  |  |  |  |  |  |  |  |  |  |  |  |

**Supplementary table S1: nasopharyngeal cycle threshold values for each infected patient**

^ Patient 1 to 7 were asymptomatic; patient 8 to 17 were symptomatic.

* A cycle threshold (Ct) value of 45 is considered to be undetectable for the virus.
